# Descending Necrotising Mediastinitis: A Case Report Illustrating a Trend in Conservative Management

**DOI:** 10.1155/2012/504219

**Published:** 2012-02-29

**Authors:** B. A. P. Jayasekera, O. T. Dale, R. C. Corbridge

**Affiliations:** ^1^Department of General Surgery, Royal Berkshire Hospital, Reading RG1 5AN, UK; ^2^The West Wing, The John Radcliffe Hospital, Oxford OX3 9DU, UK; ^3^Department of ENT, Royal Berkshire Hospital, Reading RG1 5AN, UK

## Abstract

The mortality rate from descending necrotising mediastinitis (DNM) has declined since its first description in 1938. The decline in mortality has been attributed to earlier diagnosis by way of contrast-enhanced computed tomographic (CT) scanning and aggressive surgical intervention in the form of transthoracic drainage. We describe a case of DNM with involvement of anterior and posterior mediastinum down to the diaphragm, managed by cervicotomy and transverse cervical drainage with placement of corrugated drains and a pleural chest drain, with a delayed mediastinoscopy and mediastinal drain placement. We advocate a conservative approach with limited debridement and emphasis on drainage of infection in line with published case series.

## 1. Introduction

Descending necrotising mediastinitis describes a necrotising fasciitis from an oropharyngeal or primary neck origin, with spread along cervical tissue planes into the mediastinum. Since its first description in 1938 [1], the mortality rate has declined with prompt diagnosis by way of contrast-enhanced computed tomographic (CT) scanning and aggressive surgical intervention. Whilst most reports support combined cervical and transthoracic drainage and debridement, the optimal method of drainage is contended. We describe the management of a young man with DNM with cervicotomy and transcervical mediastinal drainage with a pleural chest drain and subsequent mediastinal drain.

## 2. Case Report

A 47-year-old man presented on the general medical take with a 2-day history of sore throat, right sided pleuritic chest pain, and neck swelling. On examination this patient was systemically unwell and septic with diffuse anterior neck swelling and subcutaneous crepitations. There was, however, no discolouration of the overlying skin. There was evidence of severe periodontal and gingival disease, with reduced breath sounds on the right side of the chest, and a dull percussion note at the right lung base.

**Figure 1:**
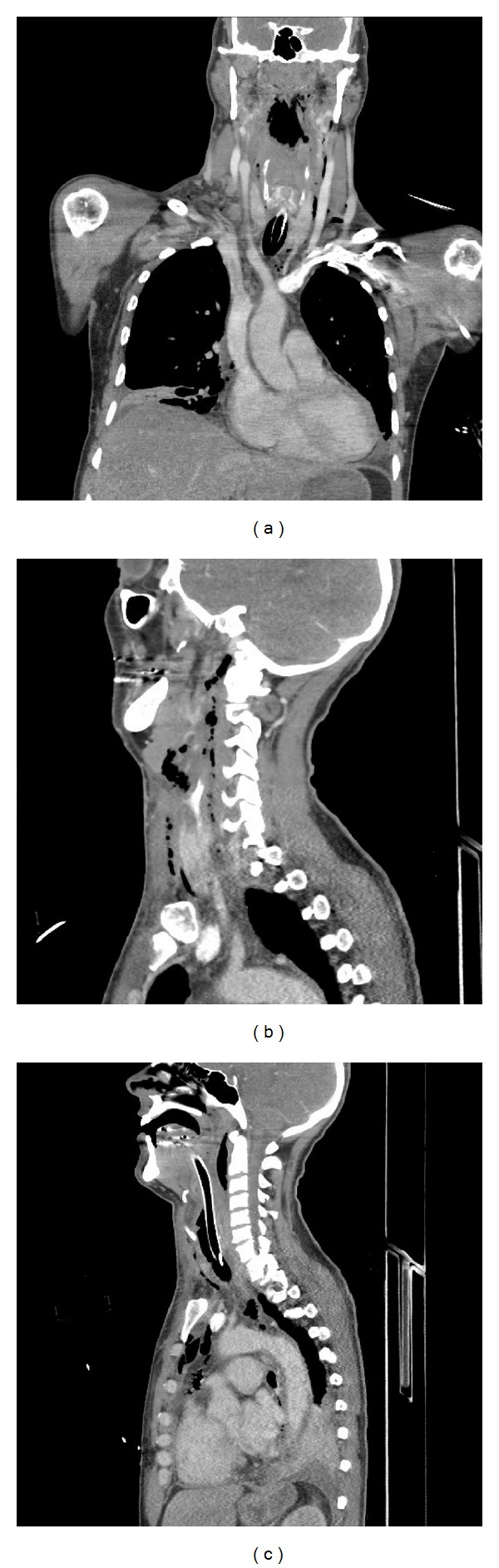
Coronal and sagittal CT scans demonstrating free gas from the skull base through the neck and into the anterior and posterior mediastinum.

Flexible nasolaryngoscopy demonstrated marked oedema of the supraglottis. The patient was intubated, and a spiral CT of the neck and chest performed (Figure 1). This showed free gas extending from retropharyngeal tissue at the skull base, through subcutaneous/intermuscular fat planes of the neck and around the larynx into the anterior and posterior mediastinum to the level of the diaphragm, with bilateral pleural effusions. No evidence of oesophageal rupture was noted.

The patient was transferred directly to the operating theatre. A bucket handle mastoid to mastoid incision was made, and subplatysmal flaps raised. This revealed a necrotic appearance of the strap muscles, thyroid tissue, and carotid sheath, with discrete pockets of pus between the tissue planes. The superficial strap muscles were debrided, and the retropharyngeal space opened, expressing more pus. Swabs were taken for microbiological culture, and all neck spaces were washed out with betadine and hydrogen peroxide solution. Corrugated drains were placed in the retropharyngeal space up to the skull base and down into the mediastinum, and the skin loosely closed.

A right-sided chest drain was inserted in the fifth intercostal space, immediately draining a litre of frank pus. The patient was then transferred to ITU.

Subsequently, a total dental clearance was performed. On ITU persistent ST elevation was noted in his ECG, with a raised troponin reflecting on going mediastinal sepsis. A further CT scan was performed showing ongoing mediastinal collections. He underwent mediastinoscopy and insertion of mediastinal drains to drain his persisting mediastinal collections. The microbiology swabs taken at the time of his initial neck debridement showed *prevotella* species and anaerobes. The patient was started on a long course of intravenous coamoxiclav. After 30 days intubated and ventilated on ITU, he was extubated and transferred to the ward to complete a 6-week course of IV antibiotics. At the time of writing the patient is GCS 15 with no residual deficits from his hospital episode.

## 3. Discussion

Various terms have been in use to describe deep spreading infections in the neck and mediastinum. The first series describing necrotising mediastinitis as a consequence of cervical suppuration was by Pearse in 1938 [1]. Of 110 patient with necrotising mediastinitis (99 from the literature and 11 from Pearse's own experience), 21 cases were oropharyngeal in origin (retropharyngeal abscess *n* = 11, peritonsillar abscess *n* = 8, Ludwigs angina *n* = 2). The majority of cases were from perforated cervical oesophagus (*n* = 64). Other aetiologies included supportive cervical lymphadenitis *n* = 13, tracheotomies *n* = 6, spondylitis of the cervical spine *n* = 3, and postthyroidectomy *n* = 3.

Estrera et al. [2] drew the distinction between descending necrotising mediastinitis (DNM) originating from oropharyngeal/deep neck infections and necrotising mediastinitis from nonoropharyngeal/cervical sources, for example, oesophagus, lung, and spine [2, 3]. They used the following criteria for diagnosis of descending necrotising mediastinitis: (1) clinical markers of severe infection, (2) characteristic radiological appearances, (3) evidence of necrotising mediastinal infection during operation or postmortem, and (4) oropharyngeal or cervical origin of descending necrotising mediastinitis.

Spread of infection is thought to occur via three principle cervical tissue spaces, namely, retropharyngeal, perivascular, and pretracheal planes, facilitated by the absence of barriers along such planes, negative intrathoracic pressure during respiration, tissue necrosis, and gas-forming organisms [3]. Wheatley et al. [4], examining all case reports published from 1960 to 1990, reported predominantly odontogenic sources of DNM. Recent meta-analyses of case series have suggested that the aetiology of DNM is predominantly from pharyngeal infections as opposed to odontogenic infections [3]. Other causes include pharyngeal perforations from foreign bodies, iatrogenic perforation, and primary neck infections. In the case described the initial infection was thought to be odontogenic in origin. The majority of infections are polymicrobial with aerobic and anaerobic bacterial species. Ridder et al. [3] identified *Streptococcus* species (*pyogenes*, intermedius, *constellatus*), as the most prevalent aerobic species in their series, with bacteroides species as the most prevalent anaerobic species.

The mortality rate has declined from 49% in the first reported case series by Pearse [1], with figures as low as 11% in some series [3]. Earlier identification with CT and more aggressive surgical drainage are thought to have contributed to the decline in mortality. Estrera et al. [2] first highlighted the value of contrast CT scans in timely identification of DNM in a patient population with often nonspecific clinical presentations. Contrast-enhanced CT scans are considered the gold standard in investigation, permitting accurate anatomical delineation of disease to guide surgical intervention. After drawing the distinction of descending necrotising mediastinitis from necrotising mediastinitis from other sources, Estrera et al. [2] questioned the adequacy of transcervical mediastinal drainage, suggesting transthoracic drainage in cases where necrosis extended below the level of the fourth thoracic vertebrae posteriorly or tracheal bifurcation. Marty-Ane et al. [5] suggested systematic transthoracic drainage in cases of DNM, irrespective of the level of mediastinal involvement. Comparing English case reports/series from 1960 to 1995, Corsten et al. [6] highlighted the favourable mortality rate in patients treated with combined surgical neck and thorax drainage compared to neck drainage alone (19% versus 47%, *P* < 0.05). They commented that thoracic drainage was often for patients who were more ill and had not responded to initial neck drainage, highlighting the merit of thoracic drainage. Brunelli et al. [7] contended the view of Marty-Ane, suggesting that transcervical drainage was adequate in cases limited to the superior mediastinum. In their series of 10 patients, Freeman et al. [8] attributed the 0% mortality rate to early diagnosis and surveillance for disease progression either by clinical suspicion or empirically at 48 to 72 hours after each operation. Any undrained collections or progression of necrosis prompted repeat surgical intervention. Mean operations per patient were higher in Freemans series than in published case reports from 1970 to 1999 (*n* = 102 patients), with more mean transcervical (4 versus 2) and transthoracic procedures (2 versus 0.7), per patient.

Endo et al. [9] classified DNM on the basis of mediastinal involvement as follows: type 1 no involvement beyond the fourth thoracic vertebrae posteriorly, and type 2a involving the anterior mediastinum, type 2b involving the anterior and posterior mediastinum. They managed type 1 cases with transcervical drainage alone (*n* = 2), type 2a cases with combined transcervicotomy and subxiphoid mediastinal drainage (*n* = 1), and type 2b cases with combined transcervicotomy and thoracotomy (*n* = 1). Karkas et al. [10], using a similar algorithm, treated 17 patients successfully depending on the level, and extent of mediastinal involvement with cervicotomy for disease limited to the above carina, and combined cervicotomy and sternotomy for anterior inferior mediastinal disease or cervicotomy and posterolateral thoracotomy for posteroinferior mediastinal disease. The transthoracic approach used to drain the mediastinal disease varies, and some groups report favourable results with video-assisted thorascopic drainage for all classes of DNM [11].

Nakamori et al. [12] felt in their experience that the principal goal of surgical intervention in cervical necrotising fasciitis, and DNM was drainage of pus, rather than surgical debridement. With this mind, they opted for percutaneous drainage of cervical necrotising fasciitis and DNM under radiological control. 6 cases of cervical necrotising fasciitis complicated by DNM were successfully managed with percutaneous mediastinal drainage.

In our case we opted for limited surgical debridement with cervicotomy and transcervical mediastinal drainage, with corrugated drain placed into the superior mediastinum. Despite the presence of posterior mediastinal involvement, we felt that a pleural chest drain would provide adequate drainage given the likely contiguity with the mediastinum. However, the patient required subsequent mediastinoscopy and insertion of a mediastinal drain for a persistent collection, highlighting the need to review the patient for persistent/spreading infection clinically or for surveillance with CT scans to identify unanticipated collections/spread of disease. We advocate a conservative approach in managing DNM and in this regard agree with Nakamori et al. that surgical drainage rather than debridement should be the principal aim of surgical intervention though the short interval from presentation to surgery would have contributed to the patient's survival.

In summary DNM is an uncommon entity with an often nonspecific clinical presentation. A high index of suspicion when managing patients with deep neck infections should prompt timely investigation in the form of contrast-enhanced CT scanning to identify DNM. Management priorities should include securing the patients airway, initiating broad-spectrum antibiotics to cover aerobic and anaerobic species, and surgical intervention with minimal delay. A multidisciplinary approach is necessary, with ENT and cardiothoracic input, and maxillofacial surgery if an odontogenic infection is suspected. If transthoracic drainage is required the choice of percutaneous, thoracoscopic, or open surgery should be guided by the facilities available and the experience of the surgical teams involved, however, where possible we advocate conservative intervention with the emphasis on ensuring adequate drainage. Postoperative surveillance for clinical signs of persistent infection or empirical CT scans should be considered.
